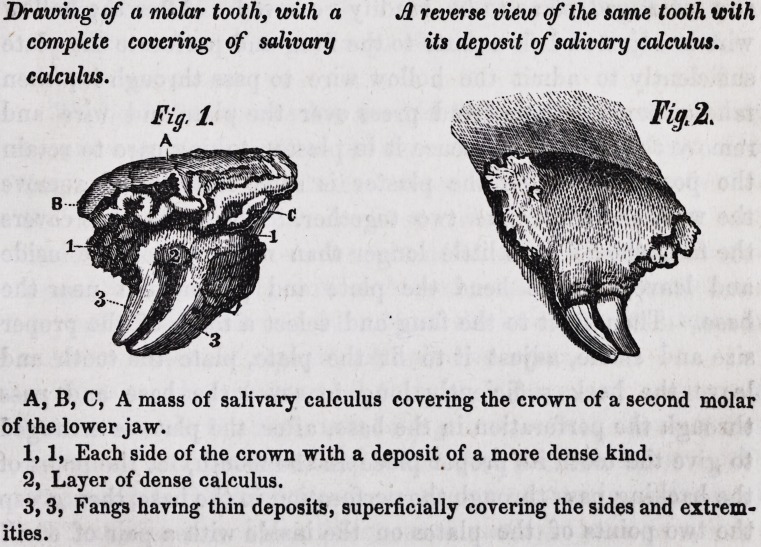# On the Large Deposits of Salivary Calculus in Gouty Persons

**Published:** 1853-01

**Authors:** J. L. Levison


### ARTICLE X.

*On the Large Deposits of Salivary Calculus in Gouty Persons. (For the Quarterly Journal of Dental Surgery.)* By J. L. LEVISON, D. D. S.

I send you a drawing from a tooth in my possession, which was discovered in the following manner: a brother of mine, when in practice some years since, at Chester, had a farmer call on him for some new teeth. On looking into his mouth, which was in a most disgusting condition, (the few front teeth being covered with what is usually called tartar,) when he observed what appeared a rudely formed block, and the following colloquy took place.

"I perceive you have had something done already?"

"No, that I aint."

"Why you have had a bone piece on this side?"

"But I say I aint." And he laughed in my brother's face, to the annoyance of his olfactory nerves.

The appearance of the supposed block, rather puzzled him, and putting his finger and thumb on it, it moved about quite loosely, and hence, without any particular effort, he removed it.

It is another confirmation of one of my earliest observed original facts,* viz. that where there exists a gouty *diathesis*,

*Some years I attended a lady who had an hereditary gouty affection, and so great was the deposit of tartar, that I removed it at the end of every month or six weeks. After two or three years it ceased. There was not any thing to indicate constitutional change, and I suspected that the kidneys had taken up the action previously performed by the salivary glands, and I subsequently ascertained such to be the case, she had large deposits of gravel.

there is generally a very great accumulation of earthy salts. Sometimes in the bladder, sometimes in the mouth, and in extreme forms of the disease when it has become chronic, large masses of phosphate and carbonate of lime are formed under the cuticle of the hands and fingers (gout and stones,) and sometimes they are coughed up from the lungs in consumptive cases. When, therefore, large deposits of the salivary calculus takes place, it may be assumed as a true diagnotic sign that the patient is either a gouty or rheumatic subject, and the constitutional and dietetical treatment should be accordingly indicated; and the local foreign matter should be carefully removed by some surgeon dentist.

***Drawing of a molar tooth, with a complete covering of salivary calculus.***

*Fig. 1.*

***A reverse view of the same tooth with its deposit of salivary calculus.***

*Fig. 2.*

**A, B, C, A mass of salivary calculus covering the crown of a second molar of the lower jaw.**

**1, 1, Each side of the crown with a deposit of a more dense kind.**

**2, Layer of dense calculus.**

**3, 3, Fangs having thin deposits, superficially covering the sides and extremities.**

*Devonshire Place, Brighton, Sept.* 16*th*, 1852.

## Figures and Tables

**Figure f1:**